# Similar treatment outcomes with *Ginkgo biloba* extract EGb 761 and donepezil in Alzheimer’s dementia in very old age: A retrospective observational study 

**DOI:** 10.5414/CP203103

**Published:** 2018-01-10

**Authors:** Michael Rapp, Martin Burkart, Thomas Kohlmann, Jens Bohlken

**Affiliations:** 1 Praxis Bohlken/Otto/Schultes-Platzek, Berlin,; 2 Dr. Willmar Schwabe Gmbh & Co. KG, Karlsruhe, and; 3 Institute for Community Medicine, University of Greifswald, Greifswald, Germany

**Keywords:** Ginkgo biloba extract, EGb 761, donepezil, dementia, Alzheimer’s disease, oldest old, treatment outcome, safety

## Abstract

Objective: To provide pilot data for the safety and efficacy of EGb 761 in the oldest-old patients (aged 80 or older). Materials and methods: In a retrospective analysis, we compared treatment outcomes with EGb 761 or donepezil over 12 months in 189 patients aged 80 years or older suffering from Alzheimer’s disease (AD). Results: Over 12 months, there was no significant difference in cognitive decline, measured with the mini-mental state examination (MMSE) score, between donepezil and EGb 761 (p = 0.31). We found more adverse events in the donepezil group. Conclusion: Results suggest similar effects on cognitive symptoms from the use of EGb 761 in the treatment of dementia in AD together with favorable safety compared to donepezil.

## Introduction 

With ongoing increases in the aging population in most societies, the majority of increases in dementia prevalence are expected to occur in people aged 80 years and older [1]. At the same time, there are relatively little data available on the safety and efficacy of pharmacological treatments for Alzheimer’s disease (AD) in this oldest-old age group. 

The clinical efficacy and safety of defined quantified *Ginkgo biloba* extract EGb 761 in the treatment of dementia has been demonstrated by a series of randomized, placebo-controlled, double-blind clinical trials (for an overview see [2, 3, 4, 5]). More than 1,000 patients included in these trials were 60 years of age or older [6]. However, the mean age was between 63 and 79 years, and studies in patients aged 80 or older have not been conducted [5]. 

In Germany, donepezil is the cholinesterase inhibitor prescribed most frequently to dementia patients aged 80 years or older [7]. However, clinical evidence of the compound’s safety and efficacy in the oldest old is limited [8]. 

Therefore, we conducted a retrospective analysis of service provision data from a neuropsychiatric practice in order to compare the safety and treatment outcome of EGb 761 to donepezil over 12 months in a sample of demented patients aged 80 years or older suffering from AD. 

## Materials and methods 

We retrospectively identified all demented AD patients treated in a neuropsychiatric practice between January 1, 2002, and December 31, 2014. The following information was extracted from medical records for a period of 12 months post treatment initiation and anonymized: gender, age in years, dementia diagnosis, scores on the MMSE (mini-mental state examination), and IADL (instrumental activities of daily living). Furthermore, we coded reasons for treatment discontinuation at 6 and 12 months, respectively, from information obtained in the medical records and categorized them as follows: (i) adverse events, (ii) patients’ wish or compliance issues, (iii) lack of clinical evidence for efficacy, (iv) other/unspecified. 

Baseline differences between donepezil and EGb 761 groups were assessed by analysis of variance and χ^2^-tests as appropriate. Differences in reasons for treatment discontinuation at 6 and 12 months were analyzed using χ^2^-tests. For the analysis of treatment effects, we computed multivariate analyses of variance with three repeated measures (baseline, 6, and 12 months) with MMSE scores as the primary outcome variable, controlling for age, gender, and IADL. For sensitivity analyses, we repeated this analysis with the full sample using the last observation carried forward (LOCF) approach. All analyses were conducted with SPSS Statistics for Windows (Version 21.0) and α set to 0.05 (two-tailed). The sample size in this pilot study was not ascertained a priori. 

The study was approved by the ethics committee of the University of Greifswald (BB 60/12), Greifswald, Germany. 

### Patients 

We included patients meeting the following inclusion criteria: presence of an ICD-10 diagnosis of dementia in AD (F00.x [9]), treatment with either donepezil or EGb 761*, aged 80 years or older, and a score on the MMSE [10] of at least 10 points. Diagnosis of AD dementia according to ICD-10 criteria was ascertained using clinical history and clinical examination, laboratory testing according to effective German consensus guidelines for the diagnosis and treatment of dementia, neuropsychological testing including either the MMSE [10] and the DemTect [11] or the CERAD neuropsychological battery [12], assessment of instrumental activities of daily living by Lawton’s eight-item scale (IADL [13]), and brain imaging. Specifically, the diagnosis of AD required a significant cognitive decline as evidenced by either an MMSE score of 23 or below, a DemTect score of 9 or below, or a score of greater than –1.5 standard deviations below the age-, gender-, and education-specific norm on the CERAD battery [10, 11, 12] together with a significant impairment in IADL [13]. Furthermore, it required the absence of a reversible cause of dementia as evidenced by laboratory testing as well as the presence of significant temporal lobe atrophy on brain imaging, reflected by either a Scheltens index of 2 or greater, or a clinical reading of significant temporal lobe or hippocampal atrophy, in the absence of vascular lesions as evinced by the absence of a clinical reading of vascular lesions or a Fazekas score of less than 2 [14]. Patients in the donepezil group were either prescribed 5 mg (N = 76) or 10 mg (N = 20) of donepezil, and patients in the ginkgo group were uniformly prescribed 240 mg of EGb 761. For further analyses, dosage was coded dichotomously as full dose, reflecting 10 mg of donepezil and 240 mg of EGb 761, or half dose, reflecting 5 mg of donepezil. Patients were regularly followed-up at least every 6 months, including these scales. 

*EGb 761^®^ is the active ingredient of Tebonin^®^, Dr. Willmar Schwabe, Karlsruhe, Germany. 

## Results 

189 patients met the inclusion criteria. At baseline, patients treated with EGb 761 (N = 93) did not differ from patients treated with donepezil (N = 97) with respect to age (donepezil: 84.17 ± 3.66 years; EGb 761: 84.40 ± 3.31) years), gender (donepezil: 69 female; EGb 761: 69 female), MMSE (donepezil: 21.68 ± 3.13 points; EGb 761: 22.67 ± 4.00 points), and instrumental activities of daily living (donepezil: 3.35 ± 2.54 points; EGb 761: 4.17 ± 2.82 points). 

Over 12 months, a total of 94 patients discontinued treatment, 58 (30.7%) after 6 months, and another 36 (19.0%) after 12 months. There was no significant difference between the donepezil and EGb 761 group χ^2^ = 1.54, df = 2, p = 0.463). Similarly, discontinuation due to perceived lack of clinical efficacy did not differ between the two groups (χ^2^ = 0.25, df = 1, p = 0.615). However, the number of patients who discontinued due to side effects was larger in the donepezil (n = 7) as compared to the EGb 761 group (n = 1; χ^2^ = 4.51, df = 1, p < 0.05). Reasons for discontinuation are given in Table 1. 

**Figure 1.**
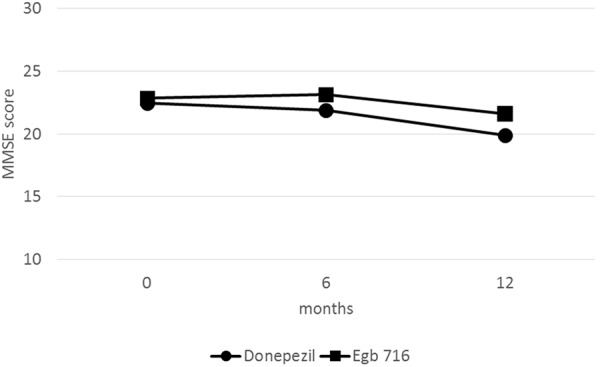
Mini-mental status examination scores over time in demented Alzheimer’s disease patients 80 years or older treated with donepezil or *Ginkgo biloba* extract EGb 761. No significant time-by-group interactions were found in repeated measures analysis of variance.

Repeated measures analysis of variance showed a significant effect for group (F_1,88_ = 5.05, p < 0.05), but not time (F_2,88_ = 2.35, p = 0.099), and no time-by-group interaction (F_2,88_ = 1.17, p = 0.314) on scores on the MMSE. Using LOCF, a comparable pattern emerged, with a significant effect for group (F_1,182_ = 7.77, p < 0.05), but not time (F_2,182_ = 2.64, p = 0.073), nor a time-by-group interaction (F_2,182_ = 1.78, p = 0.170) on scores on the MMSE. Results did not change when we added dosage (coded dichotomously as full dose versus half dose) as covariate. Numerically, these results suggested a favorable treatment effect in the EGb 761 group. Mean scores on the MMSE in both groups are depicted in Figure 1. 

## Discussion 

In this retrospective analysis, we found no significant differences with respect to treatment outcome as measured with the MMSE over 12 months in demented AD patients aged 80 or older between *Gingko biloba* extract EGb 761 and donepezil. LOCF analyses suggested a mild favorable effect for EGb 761 over donepezil. We did indeed find that discontinuation was comparable over 6 months. We found that discontinuation in the donepezil group more often occurred due to adverse events, suggesting a more favorable safety profile for EGb 761. 

The discontinuation rates were relatively high compared to randomized clinical trials; however, in light of recent service provision data from larger samples in Germany, discontinuation rates of 50% or higher over 12 months seem to be rather common in clinical practice [15, 16]. 

Obviously, retrospective data analyses are flawed by the lack of blinded random assignment. Furthermore, this was a monocentric study, and it is an open question to what extent results can be generalized to other settings. In addition, we were only able to follow up on patients using the MMSE, and lacked longitudinal data on activities of daily living (ADLs)****and other measures of change. Furthermore, the majority of patients did not receive a full dose of donepezil, while all patients in the EGb 761 group did receive a full dosage. While this finding may represent an effect of adverse events in itself, we cannot rule out that the current absence of a difference may be due to a less effective dose in the donepezil group. On the other hand, our study may provide useful data for the practicing clinician and may inspire future randomized controlled trials in the oldest old. 

## Conclusion 

We found comparable treatment effects on cognitive symptoms of *Gingko biloba* extract EGb 761 and donepezil for the treatment of cognitive symptoms in demented AD patients aged 80 years or older, and a more favorable safety profile. 

## Acknowledgment 

We thank Ms. Verena Huwe for help with data extraction. 

## Funding 

See conflict of interest. 

## Conflict of interest 

M.A. Rapp received a honorarium from Dr. Willmar Schwabe GmbH & Co. KG for preparing the first draft of this manuscript. M.A. Rapp also received consultant fees from Lilly, Inc., and speaker honoraria from Merck, Glaxo Smith Kline, Servier, and Johnson and Johnson. J. Bohlken received grant support from Dr. Willmar Schwabe GmbH & Co.KG for research studies, and consultant fees from Lilly, Inc. 

**Table 1.** Reasons for discontinuation at 6 and 12 months, respectively, as derived from clinical records. Data indicate N (% of total population on respective drug).

	Donepezil (N = 96)		EGb 761 (N = 93)	
	6 months	12 months	6 months	12 months
Adverse events	3 (3.13)	4 (4.17)	1 (1.08)	0
Patients’ wish or compliance issues	1 (1.0)	0	5 (5.38)	7 (7.53)
Lack of clinical evidence for efficacy	16 (16.67)	11 (11.46)	12 (12.90)	11 (11.83)
Unspecified	10 (10.42)	0	10 (10.75)	3 (3.23)
